# Primary Malignant Meningeal Melanoma Complicated by Cerebral Venous Sinus Thrombosis: An Illustrative Case With a Systematic Review of the Literature

**DOI:** 10.7759/cureus.66710

**Published:** 2024-08-12

**Authors:** Jennifer A Mann, Dhyey Bhatt, Michael K Tso

**Affiliations:** 1 Department of Clinical Neurosciences, University of Calgary, Calgary, CAN; 2 Neurosurgery, Kelowna General Hospital, University of British Columbia, Kelowna, CAN

**Keywords:** melanoma, rare melanoma, primary leptomeningeal melanoma, primary brain tumors, meningeal neoplasms, meningeal melanoma, primary intracranial melanoma, primary malignant meningeal melanoma

## Abstract

Melanocytic tumors of the central nervous system (CNS) such as meningeal melanoma are exceedingly rare tumours derived from leptomeningeal melanocytes. We report an illustrative case of a previously healthy 47-year-old male who presented with tonic-clonic seizure. Magnetic resonance imaging (MRI) with contrast demonstrated a homogenously enhancing right temporal extra-axial lesion. The patient was stabilized on anti-epileptic medications and dexamethasone prior to proceeding with complete surgical resection of the lesion. Intraoperatively, the lesion was heavily pigmented with invasion of the surrounding dura and skull. Histopathology revealed a poorly differentiated neoplasm with nuclear atypia and melanin-containing cells with strong SOX10 and variable S100 positivity. Computed tomography (CT) of the chest, abdomen, and pelvis showed no metastatic disease, and molecular profiling was negative including absent BRAF mutation. He began checkpoint inhibitor therapy and subsequently developed cerebral venous sinus thrombosis managed with anticoagulation. Sixteen months post-operatively, he was neurologically intact, working full-time, and had resumed immunotherapy.

We systematically reviewed the literature on primary intracranial malignant melanoma (PIMM) with the goal of understanding the prognosis and best treatment options for this disease. Our systematic review produced 82 articles (118 unique cases) of PIMM. The average age at diagnosis was 45.9 years (95% CI:42.9-48.9), and headache (54.2%) was the most common initial presentation. Eighty-nine percent of patients had primary surgical resection, and 41.0% of these individuals experienced a recurrence with a mean time to recurrence of 19.6 months (95% CI:6.95-32.23). Adjuvant therapy was administered in 65.7% of surgically resected patients; including radiotherapy, chemotherapy, immunotherapy, or a combination. In summary, PIMM is a rare tumour that can appear radiographically similar to meningioma. The results of our systematic review demonstrate that surgical resection remains the mainstay of therapy for best long-term prognosis.

## Introduction

Primary malignant meningeal melanomas are exceptionally rare intracranial tumors with an annual incidence of approximately 0.5 per 10 million [1]. Thought to have been first described by Virchow in 1859 [2], primary melanocytic tumors of the central nervous system (CNS) are a group of lesions ranging from benign to malignant and are derived from melanocytic cells. The 2021 WHO classification differentiates melanocytic tumors into diffuse meningeal melanocytic neoplasms (meningeal melanocytosis and meningeal melanomatosis), and circumscribed meningeal melanocytic neoplasms (meningeal melanocytoma and meningeal melanoma) [3].

Embryologically, primary melanocytic tumors are derived from CNS melanocytes which arise from multipotent neural crest cells [4]. Pathologically, malignant melanomas differ from more benign melanocytic tumors of the CNS due to their epithelioid and spindled cells with significant cellularity, pleomorphism, and nuclear atypia, often with CNS invasion or necrosis [4,5].

Primary melanomas of the CNS can occur anywhere in the neuroaxis. Radiographically, they appear as T1 hyperintense lesions and T2 hypointense lesions, pertaining to their melanin content, and they tend to enhance [4,6]. Often, primary meningeal melanomas mimic the radiographic appearance of meningiomas [2]. Clinically, primary meningeal melanomas present with typical features of intracranial mass lesions including intracranial hypertension, focal neurological deficit, or seizure [6].

We report a systematic review of primary malignant meningeal melanoma, alongside an illustrative case of a 47-year-old male who presented with seizure and was diagnosed with a right temporal lesion that was amenable to primary surgical resection. To our knowledge, there are no discrete guidelines to dictate the standard of care for management of primary intracranial meningeal melanoma (PIMM). Accepted management consists of maximal safe resection and/or radiotherapy, with a variety of chemotherapeutic agents trialed [2,6].

## Case presentation

Case presentation

**Figure 1:**
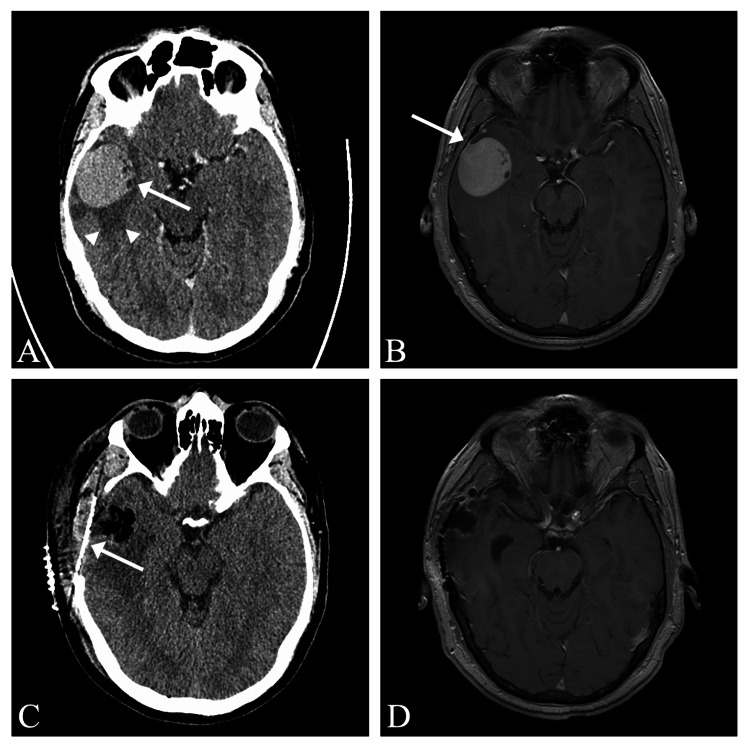
Pre- and post-operative neuroimaging A) Contrast-enhanced CT head showing a well-circumscribed right temporal lesion (white arrow) with homogenous contrast enhancement and mild associated edema (white arrowheads delineate edema); B) T1W post-gadolinium MRI showing a right temporal lesion with homogenous contrast enhancement and dural attachment (white arrow); C) Post-operative CT head showing gross total resection of the lesion with titanium mesh cranioplasty in place (white arrow); D) T1W post-gadolinium MRI three months post-operatively showing no evidence of residual or recurrent enhancing disease.

We present the case of a 47-year-old male who presented to our tertiary care centre (Kelowna General Hospital) due to a generalized tonic-clonic seizure. This was an otherwise healthy patient with no diagnosed medical conditions and no prior history of seizure. Computed tomography (CT) head demonstrated a round extra-axial mass lesion in the right anterior temporal area with well-defined borders, homogenous contrast enhancement, and moderate associated vasogenic edema (Figure 1A). This was further assessed with magnetic resonance imaging (MRI) of the brain which demonstrated a homogenously enhancing extra-axial lesion in the right anterior temporal area and what appeared to be an enhancing dural tail (Figure 1B).

**Figure 2:**
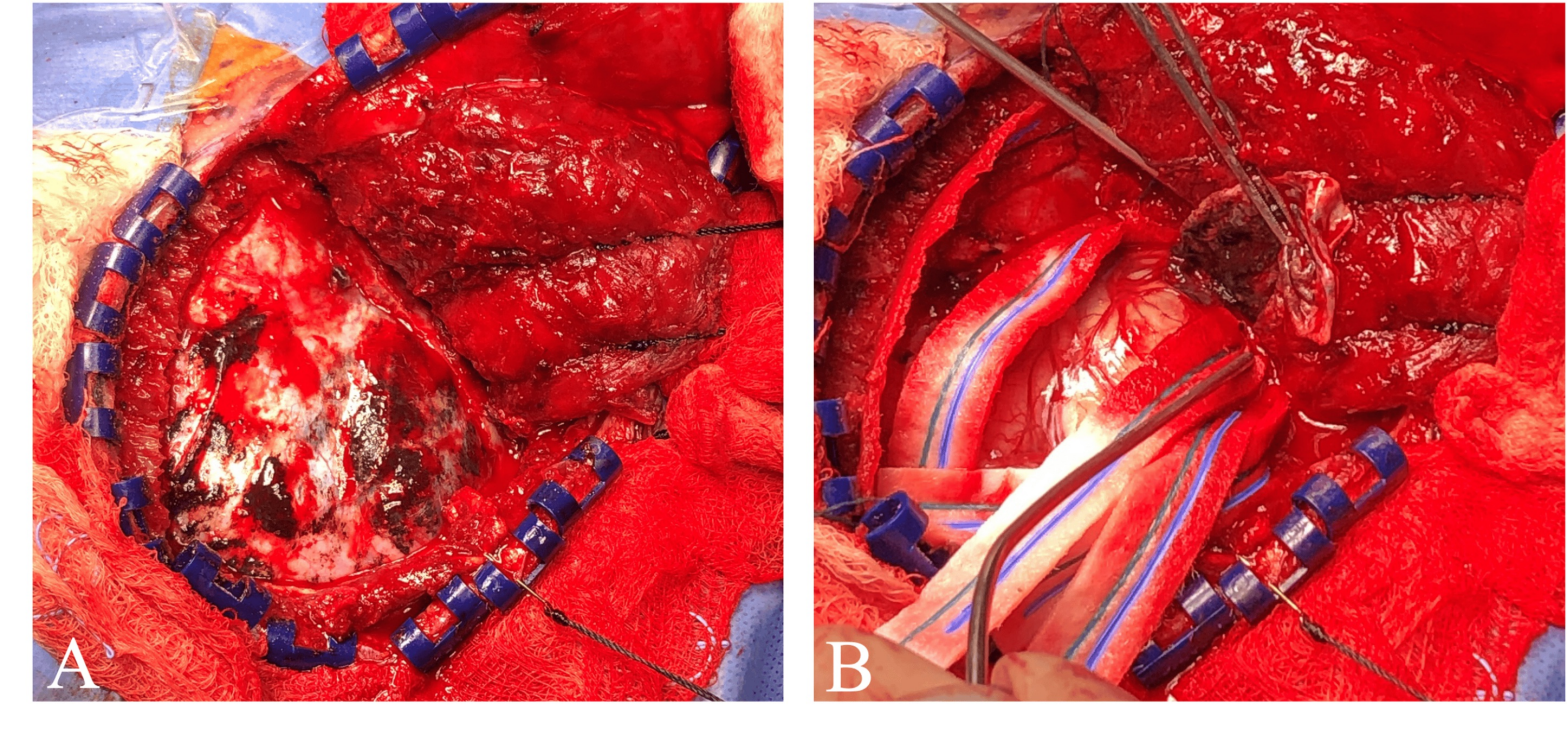
Intraoperative images A) The dura mater overlying the lesion following craniectomy is visible displaying melanin staining; B) Diffuse melanin staining and lesion invasion in the subdural space

The patient was initially treated with levetiracetam and dexamethasone and was discharged home following stabilization and a two-day hospitalization. Approximately one month later the patient returned for elective surgery and underwent a right temporal craniectomy for resection of the lesion. Upon removal of the bone flap, it was apparent that there was a pigmented lesion invading the dura and skull, with melanin-staining of the diploic space and diffusely throughout the dura (Figure 2). Due to dural infiltration, the dura was resected following gross total removal of the lesion. The bone flap was not replaced and a titanium mesh cranioplasty was performed (Figure 1C, 1D). The patient had an uncomplicated post-operative course and was discharged home on post-operative day two. 

Pathology revealed strong positivity for SOX10 and variable S100 positivity, supportive of the diagnosis of melanoma. The patient was assessed by Ophthalmology and there was no evidence of ocular melanoma. He had a positron emission tomography (PET) scan which revealed no other foci of metastatic disease. He started on his first cycle of ipilimumab and nivolumab checkpoint inhibitor therapy, which was complicated by toxicities including colitis, thyroiditis, and acute kidney injury, and subsequently managed with steroids to good effect. 

He re-presented to hospital seven weeks post-operatively with headache and right-hand clumsiness. CT/CTA showed extensive cerebral venous sinus thrombosis (CVST) involving the superior sagittal sinus, left transverse sinus, left sigmoid sinus, and left internal jugular vein into the vein of Trolard and cortical veins with an acute venous infarct involving the left precentral gyrus hand knob region. Anticoagulation with intravenous heparin (no bolus) was initiated and he was monitored in the Intensive Care Unit. He remained neurologically stable and transitioned to oral anticoagulation (dabigatran). At last follow-up 16 months later, he had minimal right-hand clumsiness and MRI revealed significant improvement in his venous sinus thrombosis with recanalization of the left jugular, transverse, and sagittal sinuses. The patient has since returned to work full-time, resumed immunotherapy, and did not receive any radiation therapy. 

Systematic review 

**Figure 3:**
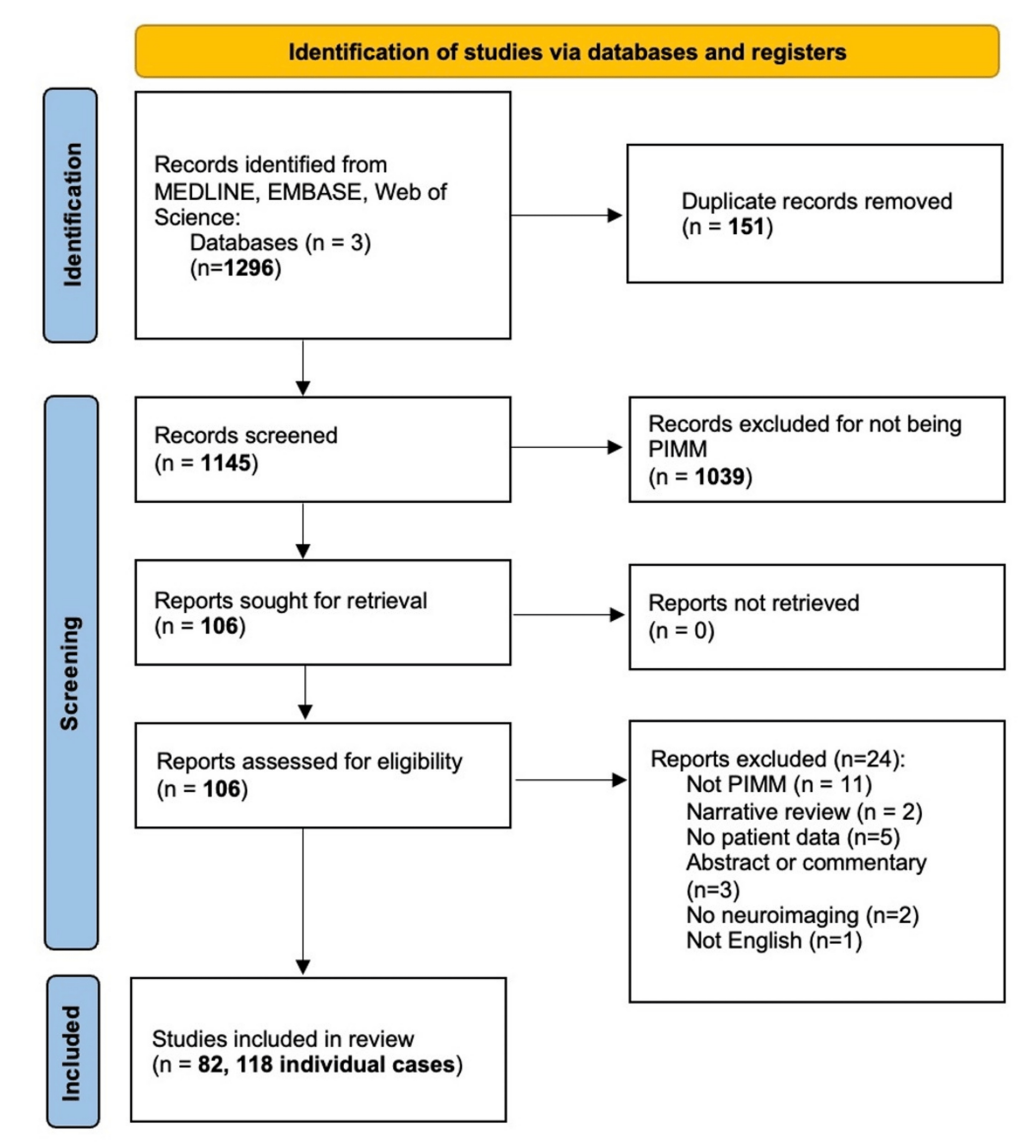
Study selection flow chart PIMM: primary intracranial malignant melanoma

We performed a systematic review guided by the standards of the Preferred Reporting Items for Systematic Reviews and Meta-Analyses (PRISMA) statement [7]. To perform a thorough review of the literature, we queried MEDLINE, EMBASE, and Web of Science for full-text English language publications of primary intracranial meningeal melanoma from inception to May 1, 2022. Further details are available in our search strategy (Table 1) and study selection flow chart (Figure 3). We excluded pediatric cases and primary melanomas of the spine to create a homogenous cohort of adult patients. We excluded cases associated with nevus of ota, congenital melanocytic naevi, and neurocutaneous melanosis due to the premalignant potential of these conditions and possible association with metastases. Two authors (JAM, DB) independently screened titles, abstracts, and full texts to identify cases of PIMM. Data extraction was performed by two authors (JAM, DB) independently, and the following information was collected: patient sex, age, clinical presentation, radiographic lesion location, initial management, adjunctive therapies, time to recurrence and/or death, and total follow-up duration. Any missing or unclear information was coded as missing data and excluded from final statistical analysis which is presented tabularly and summarized descriptively. 

**Table 1:** Search Strategy CNS: central nervous system

Search number	Search query
1	meningeal melanoma.mp
2	primary intracranial melanoma.mp
3	primary central nervous system melanoma.mp
4	primary CNS melanoma.mp
5	1 or 2 or 3 or 4
6	limit 5 to (English Language)
7	5 and 6

Our systematic review identified 1145 unique records, from which 106 full texts were reviewed and 82 met inclusion criteria (Figure 3) [1,2,5,8-86]. These 82 articles presented 118 individual cases of primary intracranial meningeal melanoma. The average age at diagnosis was 45.9 years (95% CI 42.9-48.9) with a slight female preponderance (57%, 67/118). The most commonly documented clinical presentations included headache (54.2%, 64/118), focal neurological deficit (24.6%, 29/118), nausea/vomiting (22.0%, 26/118), seizure (10.2%, 12/118), gait disturbance (8.5%, 10/118), and visual disturbance (7.6%, 9/118). Two or more of these presentations were reported in 61.9% (73/118) of individuals. Tumor location dictated by neuroimaging included frontal lobe (26.3%, 31/118), parietal lobe (26.3%, 31/118), temporal lobe (18.6%, 22/118), skull base (13.6%, 16/118), pineal gland (8.5%, 10/118), and occipital lobe (5.9%, 7/118). The most common approach was primary surgical resection of the tumor in 89.0% (105/118) of individuals, and 41.0% (43/105) of these individuals had a reported recurrence with a mean time to recurrence of 19.6 months (95% CI 6.95-32.23) based on follow-up from 40 individual cases. Repeat surgical resection occurred in 6.7% (7/105) of individuals who underwent primary surgical resection. The average follow-up length was 24.7 months (95% CI 18.1-31.3), based on reported values from 40 individuals. Mortality was reported in 36.4% (43/118) with an average time to death of 18.9 months after initial resection (95% CI 8.00-29.88). Radiation therapy (RT), chemotherapy (CT), or immunotherapy use was reported in 61.0% (72/118) of individuals, with three patients receiving RT or CT as their primary treatment modality, and 69 patients undergoing adjuvant therapy following their initial surgical resection. Of individuals receiving adjuvant therapy, 59.4% (41/69) had radiotherapy alone (including whole brain radiotherapy (WBRT), stereotactic radiosurgery (SRS), or gamma knife), 5.8% (4/69) had chemotherapy alone, 2.9% (2/69) had immunotherapy alone, and 31.9% (22/69) had a combination of adjuvant therapies. 

## Discussion

Primary intracranial malignant melanoma is a rare CNS tumor amenable to primary surgical resection with a variable post-operative course. We presented the case of a patient with PIMM managed with primary surgical resection followed by adjuvant immune checkpoint inhibitor (ICI) therapy. While the patient had a good post-surgical outcome, his course was complicated by immunotherapy toxicities and CVST, a complication that was not documented in any of the cases we encountered in our systematic review. This is likely due to short follow-up time (or no follow-up) reported in case reports and case series, and variable use of adjuvant therapies. In our patient, CVST was most likely the result of his cancer, or the checkpoint inhibitors he was actively being treated with. Roopkumar and colleagues have described that the use of immune modulators such as ipilimumab and nivolumab for cancer immunotherapy has a 24% incidence of venous thromboembolism (VTE) [87]. However, it must be acknowledged that active cancer is a well-known risk factor for VTE on its own. Cancers such as melanoma induce a hypercoagulable state that has been associated with VTE, including CVST [88]. Sussman and colleagues found that in patients being treated for melanoma, while 16.2% of patients experienced VTE, 9.3% had the event after initiation of ICI therapy, the incidence of which was particularly high in combination ICI therapy [89]. Many thromboembolic events, whether associated with cancer or ICI therapy, are reported to be in deep veins and pulmonary structures at presentation, and CVST is a particularly rare complication among VTE events that commonly occur in these contexts. Additional etiologic considerations include that CVST may be the result of malignant sinus invasion, or as a consequence of surgical intervention. The contralateral localization makes malignant invasion less likely, and the timeline of CVST occurrence seven weeks post-operatively makes the operative intervention in this case an unlikely contributor as post-operative sinus thrombosis tends to be fairly immediate. Thus, in our patient it is most likely that the hypercoagulable state induced by malignancy, plus or minus microscopic neoplastic invasion of the sinus, alongside the added contributor of adjuvant ICI therapy resulted in the unfortunate consequence of CVST. 

While our patient made a full neurological recovery, CVST has the potential to cause neurologic morbidity that makes its prevention and surveillance imperative. Therefore, we use this case to draw attention to the need for adequate risk assessment and surveillance for those with PIMM being treated with any adjuvant therapies, particularly immune checkpoint inhibitors, to avoid potential complications.

We conducted a systematic review as we sought to better understand the management and prognosis of this rare disease. The review elucidated that, despite presenting heterogeneously, the most effective initial management of PIMM is primary resection (as performed in 89.0% of cases), although this is backed by case reports and case series without any evidence from clinical trials. We found an average life expectancy of about 20 months from diagnosis, a number which should be interpreted cautiously given the heterogenous treatment approaches, use of case reports and case series alone, and small sample who was followed to death (n=43). Solitary radiotherapy was the most common type of adjuvant therapy with 59.4% of individuals receiving this therapy. However, within this category of treatment, there was heterogeneity in modality of treatment, ranging from WBRT to SRS. The immunotherapy and chemotherapy administered varied widely, with many different agents trialed (e.g. ipilimumab, pembrolizumab, temozolomide, procarbazine lomustine and vincristine (PCV), methyl-CCNU) [9,39,42,44,46,57], and the mode of administration including both systemic and intrathecal [18]. The optimal adjuvant therapy for treatment of PIMM remains unclear due to lack of comparative data and the overall rarity of this disease. This data reveals the need for further study of adjuvant therapy for PIMM to optimize patient care and further improve prognosis for these individuals.

## Conclusions

Primary malignant meningeal melanoma is a rare tumor of the CNS. The results of our systematic review support initial surgical resection but do not elucidate which adjuvant treatment strategy is most effective for these patients. We found a mean time to recurrence post-operatively of about 20 months, an estimate which can aid with patient counselling and management of expectations. We described an illustrative case treated with primary surgical resection and adjuvant ICI. We describe this case to demonstrate a straightforward treatment course with excellent functional and radiographic outcome, and to describe effective treatment of the complication of CVST.
